# Development, Validation, and Stability Assessment Application of RP-HPLC-DAD Method for Quantification of Ampicillin in Total Parenteral Nutrition Admixtures

**DOI:** 10.3390/antibiotics8040268

**Published:** 2019-12-15

**Authors:** Maciej Stawny, Aleksandra Gostyńska, Katarzyna Dettlaff, Anna Jelińska, Marta Kościelniak, Magdalena Ogrodowczyk

**Affiliations:** Department of Pharmaceutical Chemistry, Poznan University of Medical Sciences, 6 Grunwaldzka, 60-780 Poznań, Poland; gostynska.aleksandra@spsk2.pl (A.G.); dettlaff@ump.edu.pl (K.D.); ajelinsk@ump.edu.pl (A.J.); mkoscielniak@gmail.com (M.K.); mogrodo@ump.edu.pl (M.O.)

**Keywords:** total parenteral nutrition, ampicillin, HPLC-DAD, validation

## Abstract

Background: The administration of total parenteral nutrition (TPN) is a common procedure in intensive care units, where the concomitant use of other intravenous medication is frequently needed. One of the particularly dangerous complications for neurosurgical patients is meningitis, for which high doses of ampicillin (AMP) are used. In such cases, the addition of AMP to TPN admixtures would be a desirable procedure. Thus, the AMP determination method in TPN admixture was developed and validated. Methods: An isocratic HPLC analysis was performed on a LiChrospher C18 end-capped column (250 mm, 4.6 mm, 5 µm) with a C18 pre-column (LiChrospher 100, 4 mm, 5 µm). The flow rate was 1.0 mL min^−1^ and the detection wavelength was 230 nm. System suitability parameters, such as capacity factor, numbers of the theoretical plate, asymmetry factor, as well as validation parameters, including method precision, accuracy, linearity, selectivity, and robustness, were set up. Results: The method was shown to be linear, precise, accurate, specific, and robust, and it can be used for the quantitative analysis of AMP in TPN admixtures. Conclusions: The degradation of AMP in the TPN admixtures occurred according to first order kinetics. The degradation rate was high and dependent on the composition of the mixture and the storage conditions (t_0.5_ varied from 142.44 h to 300.45 h).

## 1. Introduction

Ampicillin ((*2S,5R,6R*)-6-[[(*2R*)-2-amino-2-phenylacetyl]amino]-3,3-dimethyl-7-oxo-4-thia-1-azabicyclo[3.2.0]heptane-2-carboxylic acid) (AMP) is well-known β-lactam antibiotic that belongs to the semi-synthetic penicillin with a wide range of activity. AMP, like other β-lactam antibiotics, due to structural similarity, interacts penicillin-binding proteins, the enzymes, which are essential for cell wall synthesis. AMP interferes final stages of the synthesis of peptidoglycan, which is the main ingredient of cell wall structure, leading to bacteria death [1,2]. AMP has bactericidal activity against both Gram-positive and Gram-negative bacteria. The drug is used in digestive, respiratory, biliary, acute, and chronic urinary tract infections. In high doses, the AMP is used in intensive care patients that suffer from meningitis, in the treatment and prevention of endocarditis and sepsis that are caused by Gram-negative *enterobacteria* [2]. In critically ill patients, the administration of AMP very often is simultaneously provided to total parenteral nutrition (TPN) admixture.

The co-administration of drugs and TPN admixtures is an important clinical problem. In recent years, this subject is being increasingly undertaken, and the results that were obtained in those studies can be applied in clinical practice [3,4,5,6,7]. Most of the researchers undertake the problem of the compatibility of drugs with TPN admixtures to determine the possibility of their simultaneous administration via Y-site to the same vascular access. For this purpose, it is evaluated whether the studied TPN admixture undergoes any changes in the pH, osmolarity, zeta potential, and particle size of the lipid emulsion. It seems reasonable to determine not only the parameters characterizing the TPN admixture, but also changes in the content of the added drug, because TPN admixture might affect its stability, leading to degradation [8].

TPN admixture is the most complex drug that is used in modern medicine. It is compounded by combining the aqueous phase (amino acids, glucose, electrolytes) with a lipid emulsion, which results in the formation of an oil-in-water emulsion. The pH of TPN admixture and the presence of mono-, di-, and trivalent ions can have a significant impact on the stability of many drugs, which results in their accelerated decomposition. The co-administration of TPN admixture with a drug to the patient without testing the stability might result in many dangerous situations, such as embolism due to the formation of insoluble particles, lack of pharmacological effect due to the inactivation of the drug or the toxic effect as a result of the formation of degradation products. This is particularly dangerous for critically ill patients, in whom TPN admixtures are very often administered for maintaining adequate nutritional status and alleviating inflammatory reactions. Such patients also belong to a group of patients receiving many drugs at the same time and who are particularly endangered by an incompatibility of TPN admixture and the drug [8,9,10,11].

The aim of the study was to develop and validate the determination method of AMP in a standard TPN admixture (ST). The developed and described below method allowed us to perform the stability studies of AMP in three different TPN compositions: high energetic (HEN), high electrolytic (HEL), and low electrolytic (LEL). In addition, we used this method for the determination of AMP in two other TPN compositions [12].

## 2. Results

**Figure 1:**
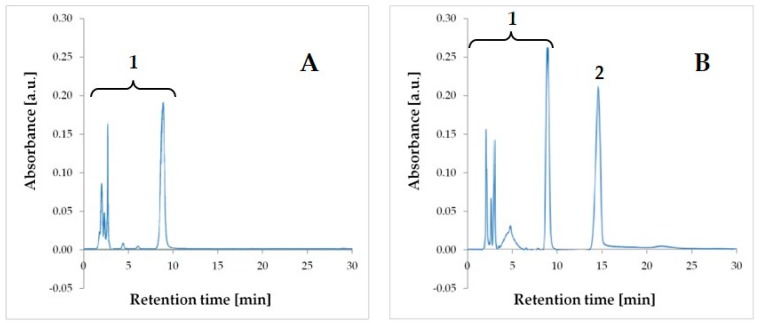
HPLC chromatograms of total parenteral nutrition (TPN) (ST composition) without AMP (**A**) and TPN (ST composition) with AMP (**B**) (1-peaks from TPN ingredients, 2-peak from AMP).

The TPN admixture samples were mixing with chloroform at a ratio of 3:1, shaking, and centrifuging in order to break up the lipid emulsion. AMP, as a highly soluble in water substance, appeared in the upper aqueous phase, which was then taken for HPLC analysis. The modification of the pharmacopoeial method [13] was applied with adjusting to the conditions of the carried out tests. For the mobile phase selection, different proportions of mobile phase A and B were tested, 50:50, 80:20, 90:10, 95:5, and 98:2 (*v/v*). The best resolution and selectivity was obtained while using 95% of phase A and 5% of phase B for chromatographic analysis. Chromatographic analysis of AMP and TPN components was established for 30 min. and the retention time (t_R_) of AMP was 15.1 ± 0.5 min. (Figure 1).

Following the ICH guidelines for analytical method validation (Q2(R1)) [14], both system suitability test (capacity factor, numbers of theoretical plates, peak asymmetry, and repeatability of peak position and area) and validation, including method selectivity, precision, accuracy, linearity, and robustness were determined. The limit of detection and quantification were also calculated. As presented in Table 1, the method is suitable for this kind of analysis, because all of the obtained parameters were below the reference limits.

**Figure 2:**
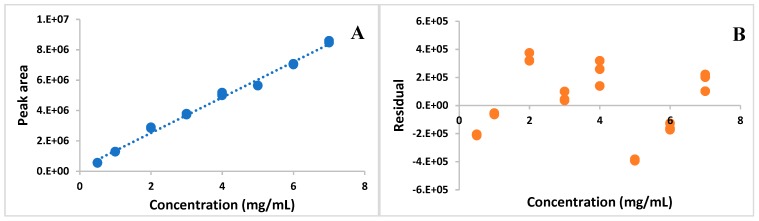
Analytical curve for AMP (**A**) and residual plotted vs. AMP concentration (**B**).

The developed HPLC method proved to be selective, because the AMP derived peak on obtained chromatograms (Figure 1) was well separated from the remaining ingredients of the TPN admixture. The method was linear in the range of 0.5–7.0 mg/mL. The relationship between the measured signal and AMP concentration that was obtained by ordinary least squares regression (OLS) was characterized by a high correlation coefficient (*r* = 0.9958) and it was described by the equation: $P_{i}=\left(11.7\pm 0.2\right)\times 10^{5}conc$ (*n* = 24), where *P_i_* is peak area and *conc* is AMP concentration (Figure 2A).

The homoscedasticity test of the data was performed in order to evaluate the fit of the chosen model (OLS). The F_calc_ (F_calc_ = 228) was found to be greater than the F_crit_, and the residues gave a wide distributed band of values around the axis of concentration (Figure 2B).

**Figure 3:**
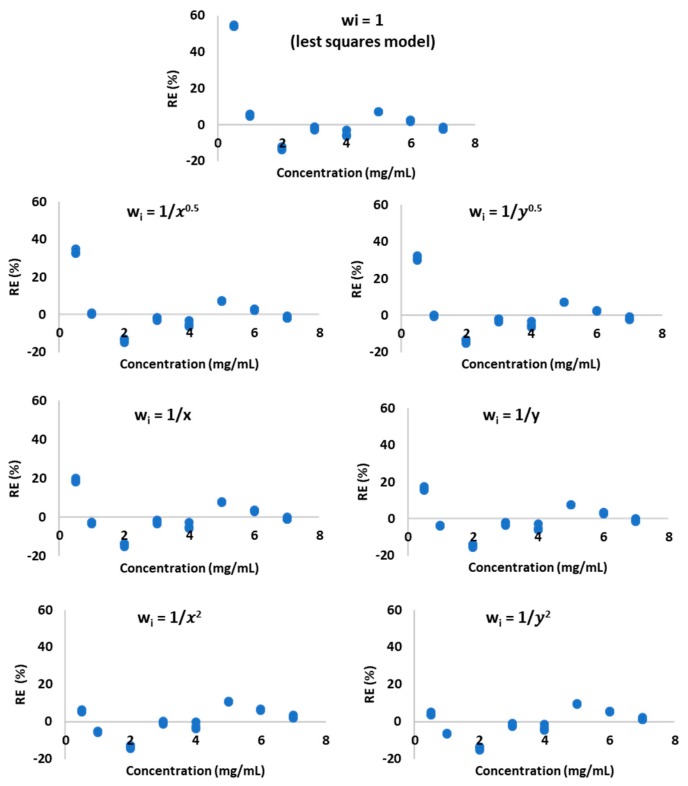
Percentage of relative errors (%RE) versus AMP concentration obtained by ordinary least squares regression (OLS) and weighted least squares regression (WLS) methods.

That evidence implied the heteroscedasticity of the data and the need for using the weighted least squares regression (WLS) to calculate the weighted regression parameters. Six empirical weights: $\frac{1}{y^{0.5}}$, $\frac{1}{y}$, $\frac{1}{y^{2}}$, $\frac{1}{x^{0.5}}$, $\frac{1}{x}$, and $\frac{1}{x^{2}}$ were used to convert the regression equation to the weighted regression equation. Table 2 provides the equation coefficients slope (a) and intercept (b). The correlation coefficient (r) was calculated for each weighting model. The best-fitting model was determined by comparing the distribution of percentage relative error (%RE) around the axis concentration of AMP (Figure 3) and the calculated values of the sums of percentage relative errors (∑%RE). The smallest ∑%RE = 2 was observed for $w_{i}=\frac{1}{y}$ (Table 2). The same model exhibited also the best %RE distribution around the concentration axis (Figure 3) and it was found to best reflect the relation between the detector signal and concentration.

The accuracy and limit of detection (LOD) and quantification (LOQ) were calculated based on OLS and WLS regression parameters. Three concentrations were selected to determine the accuracy and precision of the method: 2.0, 4.0, and 6.0 mg/mL, which corresponded to 50%, 100%, and 150% of the nominal concentration of AMP in the TPN admixtures. The value of accuracy expressed as the relative error (ε_r_) obtained by OLS was between −1.83% and −3.25% for intra-day accuracy and between −2.00% and −4.50% for inter-day accuracy. ε_r_ that was obtained by WLS ranged from -3.14% to 0.06% for intra-day accuracy and from -4.83% to – 1.42% for inter-day accuracy. The calculated relative standard deviation (RSD) ranged from 1.51% to 2.47% and from 2.17% to 2.56% for intra-day and inter-day precision, respectively. The LOD and LOQ that were calculated by OLS were 0.059 mg/mL and 0.179 mg/mL, respectively. The LOD and LOQ that were obtained by WLS were 0.057 mg/mL and 0.173 mg/mL, respectively. Table 3 presents the obtained data.

The robustness of the developed method was evaluated by changing the chromatographic conditions: the column temperature (25 ± 2 °C), the flow rate of mobile phase (1.0 ± 1 mL/min.), and the composition of the TPN admixtures (compositions ST, HEN, HEL, and LEL). Table 4 presents the results of robustness variations.

The developed and validated method was used for stability studies. We measured the AMP concentration in HEN, HEL, and LEL compositions for seven consecutive days. The content of the tested drug in TPN admixtures was determined from the obtained values of the peaks areas corresponding to AMP (Table 5). The degradation process of AMP in TPN admixtures proved to be a first-order reaction, depending on the substrate concentration described by the following equation:

$$\ln\left(P_{t}\right)=\ln\left(P_{0}\right)-\;k_{obs}\cdot t$$

where *P*_0_, *P_t_* – peak areas of AMP at time zero and time *t*, respectively; *k_obs_* – observed first-order reaction rate constant.

The semilogarithmic plots P = f(t) were linear and their slopes were equal to the rate constants of the reaction with the negative sign (−k). For these plots, the least squares method was used to calculate the parameters of the equation y = ax + b, a ± Δa, b ± Δb and the coefficient of determination (R^2^). The half-life (t_0.5_) was calculated according to the equation: $t_{0.5}=\;\frac{0.693}{k}$, where *k* is the rate constant of the reaction.

## 3. Discussion

The administration of TPN admixtures is a common medical procedure that is used to maintain adequate nutritional status in patients who cannot be orally fed. Intravenous infusions of nutritional substances often save lives, protecting patients from death resulting from malnutrition. TPN admixtures are primarily used in critically ill patients, surgical, gastroenterological, geriatric, and neurological patients. The problem of the co-administration of drugs and TPN admixtures is common, especially in intensive care units, where patients in the severe conditions received simultaneously many medications. A high dose of AMP, the first-choice drug in meningitis treatment, is frequently administrated to neurosurgical patients in intensive care units. The possibility of providing safe concomitant therapy of AMP and TPN admixture is a challenging task. Thus, the possibility of the AMP stability assessment in TPN admixtures is an interesting scientific problem.

The direct determination of AMP in TPN admixture by the HPLC method is impossible due to the presence of interfering substances and the physical properties of TPN admixture (oil-in-water emulsion). For these reasons, a new methodology that allows for the determination of AMP in TPN mixtures by HPLC has been developed in this study. The method is considered to be suitable for the determination of the drug when the validation parameters and the results that are obtained for the suitability test are within the assumed limit values. The system suitability test aimed to prove that the chromatography system is suitable and effective for the analysis. It is an integral part of the liquid chromatographic method that is used to verify that the evaluated method is able to give a good resolution between the peaks and high reproducibility of the signal in the working range. The proposed method has fulfilled all of the critical parameters of the test: capacity factor (k’ > 2.0), number of theoretical plates (*N* > 2000), asymmetry factor (As = 0.8–1.5), repeatability of t_R_ (<5%), and repeatability of peak area (<5%) (Table 1), which proves their suitability for AMP analysis in TPN admixtures [15]. The value of the asymmetry factor equal to 0.82 is low and it indicates the fronting of the peak. Nevertheless, the parameter remains in the pharmacopeial range and, thus it was accepted [15].

The validation process was performed according to the ICH Q2(R1) [14] guidelines in terms of selectivity, linearity, accuracy, precision, the limit of detection, and limit of quantification. The use of UV-DAD following chromatographic separation ensured the selectivity of this method. The observed peak of the AMP was well separated from the peaks given by the TPN admixtures ingredients. The analytical curve was prepared for eight concentrations. The minimum and maximum concentrations were 0.5 and 7 mg/mL, respectively. Plotting the peak areas vs. concentration gave a high correlation coefficient value of 0.995. However, the correlation coefficient is no longer sufficient for determining linearity, according to the literature [16,17,18]. It is suggested to evaluate the fit of the ordinary least squares linear regression by assessing the homoscedasticity of data and, in the case of heteroscedasticity search for other models, which better defined the correlation between the concentration and the response of the detector [18,19]. The analysis of the residual graphs and F-test confirmed the heteroscedasticity of data, thus the other calibration model was desirable. The best-fitting weighted linear regression model was chosen on the basis of the ∑ %RE, which was the lowest for w_i_ = $\frac{1}{y}$. The %RE values the closest to zero characterized this model.

The accuracy of the chromatography method determines the degree of deviation of the obtained values to the true values and it is expressed as ε_r_. The ε_r_ for each nominal concentration were found to be below 5% for both the OLS and WLS equation. Precision, expressed as RSD, determines the effect of random errors on the repeatability of the method. The overall RSD was below 5, which is the limit of acceptance. The standard deviation of the intercept and slope of the calibration curve are used for the calculation of LOD and LOQ of the developed method. Depending on chosen analytical curve, the LOD and LOQ were different, but not significantly. The ICH guideline Q2(R1) [14] gives five methods for determining LOD and LOQ:

- based on visual evaluation
- based on signal-to-noise
- based on the standard deviation of the response and the slope
- based on the standard deviation of the blank
- based on the calibration curve

We used the method based on standard deviation of the response and the slope, which is commonly used during the validation of analytical methods for determining the drug content in pharmaceutical preparations.

The robustness of the proposed method was determined by changing the TPN composition, column temperature and mobile phase flow rate. Changes in those parameters led to change in t_R_, P_i_, k’, number of theoretical plates, and peak asymmetry. However, the relative error of each parameter was within the acceptance criteria of 10%. We did not investigate the impact of pH during the robustness analysis. It was motivated by the use of the mobile phase, as recommended by pharmacopeia [13] for the determination of AMP and related substances, derived from the synthesis and degradation of AMP. The influence of pH of phosphate buffer was omitted, as we considered that the possible effect of pH on separation was investigated during the development of the pharmacopeial method [13].

The developed method was utilized in the stability studies. The loss of the content of AMP in different compositions of TPN admixtures during the storage time was determined. The smallest decrease of 7.85 ± 1.44% in the drug content on the first day of storage was observed for the HEN+AMP samples. However, the highest degradation was observed on the first day of storage for the TPN admixture containing reduced amounts of electrolytes (LEL + AMP), where the decrease in the drug content was 18.10 ± 3.03 %. The obtained results of AMP content analysis allow for concluding that the drug degradation in TPN admixtures depends on the composition of the TPN admixture. The stability studies of AMP allowed for determining the kinetics of the degradation of this antibiotic in TPN admixtures and finding the relationship between the composition of TPN admixtures and the stability of AMP. The degradation of AMP follows first-order kinetics.

## 4. Materials and Methods

### 4.1. Reagents, Chemical Substance, and Pharmaceutical Preparation

The AMP (purity ≥ 98%) was purchased from Sigma Aldrich (Steinheim, Germany). The acetonitrile of HPLC grade was purchased from Merck (Darmstadt, Germany). The chemical substances, potassium dihydrogen phosphate (purity: 99.5%), concentrated orthophosphoric acid, and acetic acid, were purchased from Avantor Performance Materials Poland (Gliwice, Poland). The pharmaceutical preparation Ampicillin TZF, 1000 mg, powder for solution for injection was purchased from Polfa Tarchomin S.A (Warszawa, Poland).

### 4.2. TPN Preparation

The TPN admixtures were prepared in aseptic conditions under a laminar-flow hood in accordance with the European Pharmacopoeia [19] while using a Pinnacle B. Braun automatic compounder (B. Braun Melsungen AG, Germany). Table 6 provides detailed quantity and quality composition of studied TPN. The ST composition was used for the method validation purpose, and other compositions of TPN admixtures differed in the content of electrolytes, glucose, and lipids (HEN, HEL, and LEL) were used for robustness and stability evaluation. All of the doses were calculated as one-tenth of the standard daily dose. The studied samples with the final volume of 250.0 mL were packaged in an ethylene-vinyl acetate (EVA) bags (B. Braun Melsungen AG, Germany). The tested materials were TPN admixtures with the addition of 1.0 g AMP (Ampicillin TZF 1000 mg), previously dissolved in 10.0 mL water for injection (ST + AMP, HEN + AMP, HEL + AMP, LEL + AMP) and reference sample was the standard composition of TPN admixture without the addition of the drug (ST). Each admixture was prepared separately in triplicate.

### 4.3. Development of Analytical Methodologies

The initial analytical conditions for the development of the chromatographic method for AMP were based on methods that were described in European Pharmacopoeia [13]. Two solvents were used as a mobile phase. Solvent A consisted of 0.5 mL of 12% acetic acid, 50.0 mL 0.2 M solution of potassium dihydrogen phosphate, 50.0 mL of acetonitrile, and water up to 1000 mL. Solvent B consisted of 0.5 mL of 12% acetic acid, 50.0 mL 0.2 M solution of potassium dihydrogen phosphate, 400.0 mL of acetonitrile, and water up to 1000 mL.

The standard solution of AMP was prepared by dissolving 40.0 mg of AMP in 10.0 mL of water to obtain a concentration of 4.0 mg/mL. The AMP absorption spectra were recorded at wavelengths that ranged from 200 to 400 nm. Peak identification was performed by comparing the t_R_ and absorption spectra of the samples with those of the standard solutions.

The HPLC analysis was realized while using diode array detection, by the area under the peak in the wavelength of maximal absorption 230 nm. The HPLC equipment consisted of a Merck Hitachi L-7100 HPLC pump, an L-7455 photodiode array detector, an L-7200 autosampler, a D-7000 interphase module, and a column oven set up at 25 °C. The analytical column was a reverse phase C18 (LiChrospher 100, endcapped, 5 µm) 250 mm with a pre-column reverse phase C18 (LiChrospher 100, 5 µm) 4 mm. The best chromatographic conditions were obtained while using mobile phase A and B 95:5 (v/v), flow rate 1 mL/min., and 10 μL of injection volume.

### 4.4. HPLC Samples Preparation

**Figure 4:**
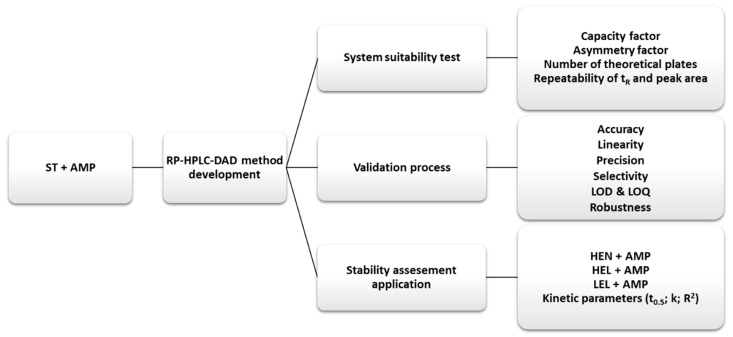
Scheme of research methodology (TPN: total parenteral nutrition admixture; ST: standard composition of TPN; AMP: ampicillin; t_R_: retention time; LOD: limit of detection; LOQ: limit of quantification; HEN: high energy composition on TPN; HEL: high electrolyte composition of TPN; LEL: low electrolyte composition of TPN; t_0.5_: half-life; k: degradation rate constant; R^2^: coefficient of determination).

The samples for HPLC analysis were prepared by mixing 3.0 mL of TPN with or without AMP with 1.0 mL of chloroform in plastic vials. The vials were shaken for 10 min on the laboratory shaker (GFL 3005, Gesellschaft für Labortechnik) and then centrifuged for 30 min. at a rate of 5800 rpm (MPW-54, MPWmed Instruments). The supernatant was collected from the centrifuged samples, filtered through a membrane filter (pore size 0.2 µm), and then injected into a chromatographic column for an HPLC analysis. Figure 4 presents the scheme of the used methodology.

### 4.5. Method Validation

#### 4.5.1. System Suitability

System suitability was examined by the determination of capacity factor (k’), the number of theoretical plates (N), and asymmetry factor (As), and was calculated according to European Pharmacopoeia [15], being calculated with the following formulas:

$$k^{\prime }=\;\frac{t_{R}}{t_{0}}-1$$

N=5.54 (tRW12)2

$$As=\;\frac{W_{5\%}}{2F}$$

where t_R_ is the retention time of the component, t_0_ is the void volume time, W12 is peak width at 50% of peak height, $W_{5\%}$ is peak width at 5% of peak height, and F is the time from the width start point at 5% of peak height to t_R._ The repeatabilities of the peak position (t_R_) and peak area were also determined.

#### 4.5.2. Linearity

The linearity of the method was established in the range of 0.5–7.0 mg/mL by diluting the AMP in TPN admixture to give the samples with following AMP concentration: 0.5 mg/mL, 1.0 mg/mL, 2.0 mg/mL, 3.0 mg/mL, 4.0 mg/mL, 5.0 mg/mL, 6.0 mg/mL and 7.0 mg/mL. These samples were prepared by weighing 12.5 mg, 25.0 mg, 50.0 mg, 75.0 mg, 100.0 mg, 125.0 mg, 150.0 mg, and 175.0 mg of AMP, transferring to 25.0 mL, dissolved in 5 mL of water, and made up to 25.0 mL with TPN admixture. Three separate series of calibration standards were prepared to establish linearity. The results were examined for a linear relationship by plotting the analyte P_i_ versus the corresponding concentrations, followed by OLS and the calculation of the slope, intercept, and coefficient of correlation.

Further calculations were performed to define the fit of the model. For this purpose, the homoscedasticity of the data while using the F-test and the residual plot was evaluated. The homogeneity of variances is proven when the calculated F-value (F_calc_) is greater than the critical F-value (F_crit_), with confidence levels of 99% for (n-1) degrees of freedom. The calibration curve was made in triplicate (the degrees of freedom were 2) and, thus, the F_crit_ was equal to 99. The F_calc_ was obtained by dividing the variance obtained for the highest concentration (S_2_) by the variance calculated for the lowest concentration of the working range (S_1_) while using the following equation: F = $\frac{S_{2}^{2}}{S_{1}^{2}}$. When the homogeneity was not found, the homoscedasticity was evaluated by plotting graphs of the residuals vs. concentration. The residuals were established by the difference between the obtained P_i_ at each point of the calibration curve and the calculated values from the OLS equation. Given the evidence of heteroscedasticity, the WLS was utilized to calculate the regression line that unifies the differences between errors throughout the working range. In the course of calculations, the appropriate weighting factors, w_i_, i.e., $\frac{1}{y^{0.5}}$, $\frac{1}{y}$, $\frac{1}{y^{2}}$, $\frac{1}{x^{0.5}}$, $\frac{1}{x}$, and $\frac{1}{x^{2}}$, were used. Each of the w_i_ was applied to the linear regression equation to obtain weighted coefficients of a (a_w_), and b (b_w_) parameters:

$$a_{w}=\frac{\sum_{i}w_{i}x_{i}y_{i}-n{\bar{X}}_{w}{\bar{Y}}_{w}}{\sum_{i}w_{i}x_{i}^{2}-n{\bar{X}}_{w}^{2}}$$

$$b_{w\;}=\;{\bar{Y}}_{w}-b\;{\bar{X}}_{w}$$

The weighted correlation coefficient (r_w_) was obtained according to the equation:

$$r=\;\frac{\sum w_{i}\;\sum w_{i}x_{i}y_{i}\;-\;\sum w_{i}x_{i}\;\sum w_{i}y_{i}}{\sqrt{\sum w_{i}\sum w_{i}x_{i}^{2\;}-\;{\left(\sum w_{i}x_{i}\right)}^{2}}\times \;\sqrt{\sum w_{i}\sum w_{i}y_{i}^{2\;}-\;{\left(\sum w_{i}y_{i}\right)}^{2}\;}}$$

Subsequently, the sums of the percentage relative errors (∑ %RE) were calculated for each model in order to determine the best fitting weighted regression line. ∑ %RE were calculated while using the equation:

$$\%RE=\frac{C{\;}_{\left(exp\right)}-C{\;}_{\left(theoret\right)}}{C{\;}_{\left(theoret\right)}},$$

where the experimental concentration, C_(exp)_ is obtained from the weighted linear equation, and C_(theoret)_ is the theoretical concentration. The regression equation that was obtained by the ordinary least squares linear regression and the best-fitting regression equation determined by the WLS were used for further calculations.

#### 4.5.3. Precision

The precision of the method was estimated by calculating the repeatability (intra-day precision) and intermediate precision (inter-day precision) by analyzing AMP at three different concentrations: 2 mg/mL, 4 mg/mL, and 6 mg/mL. The results were expressed as the RSD and calculated from the analyses of six samples for each prepared concentration. The intra-day precision was performed on the same day and another analyst repeated the inter-day precision the next day.

#### 4.5.4. Accuracy

The accuracy was performed to obtain the closeness of the agreement between the expected value and the determined value. The accuracy was tested by adding known amounts of the AMP to a known concentration of the substances. Three different concentrations were analyzed: 2 mg/mL, 4 mg/mL, and 6 mg/mL. The results were expressed as relative error (ε_r_). calculated by the relationship between the experimental concentration (C_exp_) and the theoretical concentration (C_theoret_) being expressed as a percentage:

$$\frac{C_{\left(theoret\right)\;}-\;C_{\left(\exp\right)}}{C_{\left(theoret\right)}}\;\times 100\%.$$

#### 4.5.5. Limit of Determination (LOD) & Limit of Quantification (LOQ)

The LOD and LOQ parameters were determined from the OLS and WLS equation of AMP. LOD was calculated as 3.3 *Sy/a*, and LOQ as 10 *Sy/a*, where *Sy* is a standard error and *a* is the slope of the corresponding calibration curve.

#### 4.5.6. Robustness

Robustness was evaluated by varying different method parameters to determine the reliability of the proposed HPLC method. The following parameters were changed: the composition of TPN admixture, the mobile phase flow rate, and the column temperature. For each parameter change, its influence on the t_R_, area and asymmetry of the peak, as well as the capacity factor, was evaluated by determining the relative errors of obtained results when comparing to the proposed method.

### 4.6. Stability Studies

The samples of TPN admixtures with AMP were stored at 4 ± 1 °C with light protection. The physicochemical analysis was performed on the day of the admixtures preparation and also 24 h, 48 h, 72 h, 92 h, 120 h, and 144 h after preparation. Every 24 h, a volume of 3.0 mL was withdrawn from each TPN admixture and then used for chemical stability tests.

## 5. Conclusions

The presented data showed that the developed methodology for the determination of AMP in TPN admixture possesses an application-oriented value. To the best of our knowledge, it is the first paper concerning the validation of the HPLC method that was developed for the quantification of the drug in TPN admixtures. TPN, as the complex oil-in-water emulsion, is a difficult matrix for HPLC analysis, which is why there is a need to separate the phases before the HPLC procedure. Validation was carried out, obtaining results meeting the requirements. The developed RP-HPLC-DAD method for the quantitative analysis of AMP showed to be accurate, precise, sensitive, linear, and robust.

## Figures and Tables

**Table 1:** System suitability test.

Parameter	Result	Limit
**Capacity factor (k’)**	6.51	>2.0
**Number of theoretical plates (N)**	4085	>2000
**Asymmetry factor (As)**	0.82	0.8–1.5
**Repeatability of t_R_**	1.92%	<5%
**Repeatability of peak area**	3.98%	<5%

**Table 2:** Regression parameters of the analytical curve and sums of the relative errors (Σ%RE).

Regression	w_i_	a	b	r	∑ %ER
**Ordinary least squares**	**1**	1170377	178325	0,9959	147
**Weighted least squares**	$\frac{1}{y^{0.5}}$	1187329	101887	0,9958	55
	$\frac{1}{y}$	1205581	36891	0,9954	2
	$\frac{1}{y^{2}}$	1245481	−41809	0,9925	12
	$\frac{1}{x^{0.5}}$	1187066	110362	0,9958	67
	$\frac{1}{x}$	1207040	47715	0,9954	19
	$\frac{1}{x^{2}}$	1257464	−40115	0,9937	13

**Table 3:** Accuracy and precision of the HPLC method.

Concentration (mg/mL)	Accuracy (Expressed as ε_r_) (*n* = 3) Acceptance Limit: %RE < 5%		Precision (Expressed as RSD) (*n* = 6) Acceptance Limit: RSD < 5%
	OLS $w_{i}=1$	WLS $w_{i}=\frac{1}{y}$	
**Intra-day**			
**2.0**	−2.97%	0.06%	2.47%
**4.0**	−3.25%	−3.14%	2.45%
**6.0**	−1.83%	−2.74%	1.51%
**Inter-day**			
**2.0**	−4.50%	−1.42%	2.56%
**4.0**	−2.00%	−1.93%	2.17%
**6.0**	−3.98%	−4.83%	2.45%

**Table 4:** Results of robustness variations.

Chromatographic Conditions	Robustness Factor		t_R_ (min)	Peak Area	Capacity Factor k’	Number of Theoretical Plates	Peak Asymmetry (min)
**Flow rate:** **1 mL/min** **TPN composition: ST**	**Column temperature (°C)**	23	15.44 (0.06%)	2410869 (−0.87%)	6.78 (4.15%)	4135 (1.22%)	0.83 (1.22%)
		25	**15.43**	**243203**	**6.51**	**4085**	**0.82**
		27	15.57 (0.91%)	2409197 (−0.94%)	6.92 (6.30%)	4127 (1.03%)	0.86 (4.88%)
**Column temperature: 25 °C** **TPN composition: ST**	**Flow rate** **(mL/min)**	0.9	15.15 (−1.81%)	2356539 (−3.10%)	7.02 (7.83%)	3760 (−7.96%)	0.86 (4.88%)
		1.0	**15.43**	**2432036**	**6.51**	**4085**	**0.82**
		1.1	15.84 (2.66%)	2368076 (−2.63%)	6.47 (−0.61%)	3984 (−2.47%)	0.85 (3.66%)
**Flow rate:** **1 mL/min** **Column temperature: 25 °C**	**TPN composition**	ST	**15.43**	**2432036**	**6.51**	**4085**	**0.82**
		HEN	15.33 (−0.65%)	2342875 (−3.67%)	6.45 (−0.92%)	4321 (5.78%)	0.84 (2.44%)
		HEL	15.51 (0.52%)	2401008 (−1.28%)	6.92 (6.30%)	3981 (−2.55%)	0.83 (1.22%)
		LEL	15.5 (0.45%)	2408139 (−0.98%)	7.13 (9.52%)	4123 (0.93%)	0.86 (4.88%)

The relative difference between initial value (bolded) and the value at specified condition are presented in brackets.

**Table 5:** AMP content in TPN admixtures and kinetic parameters of degradation process.

**Time** **(h)**	**HEN**	**HEL**	**LEL**
	**Mean content ± SD (%)**		
**0**	100.00 ± 1.42	100.00 ± 1.60	100.00 ± 2.07
**24**	92.15 ± 1.44	87.28 ± 2.08	81.90 ± 3.03
**48**	86.15 ± 2.15	81.38 ± 2.19	75.49 ± 2.15
**72**	83.14 ± 2.03	65.46 ± 2.82	70.70 ± 2.68
**96**	79.83 ± 2.14	59.00 ± 1.54	65.21 ± 1.36
**120**	74.71 ± 2.21	53.82 ± 2.07	61.15 ± 1.68
**144**	70.37 ± 0.92	51.67 ± 2.88	56.91 ± 0.47
**Kinetic parameters**			
**R^2^**	0.9266	0.9488	0.9573
**k (h^−1^)**	0.0023	0.0049	0.0036
**t_0.5_ (h)**	300.45	142.44	192.29
**t_0.5_ (days)**	12.52	5.93	8.01

R^2^—coefficient of determination, k—degradation rate constant, t_0.5_—half-life.

**Table 6:** Composition of studied TPN admixtures.

Ingredients	Pharmaceutical Preparation (Manufacturer)	Unit	Composition of TPN			
			ST	HEN	HEL	LEL
**Amino acids**	Aminoplasmal B. Braun 10% E (B.Braun Melsungen AG, Germany)	mL	600	600	600	600
**Carbohydrates**	40% Glucose B. Braun (B.Braun Melsungen AG, Germany)		550	650	550	550
**Lipids**	Lipofundin MCT/LCT 20% (B.Braun Melsungen AG, Germany)		300	350	300	300
**Water**	Aqua pro iniectione (B.Braun Melsungen AG, Germany)		902	752	838	935
**Sodium**	Natrium Chloratum 10% (B.Braun Melsungen AG, Germany)	mmol	102	102	136	68
**Potassium**	Kalium Chloratum 15% WZF (WZF Polfa S.A., Poland)		80	80	120	70
**Calcium**	Calcium gluconate 10% (Added Pharma, Netherlands)		5	5	7	5
**Phosphates**	Glycophos (Fresenius Kabi AB, Sweden)		24	24	36	18
**Magnesium**	Inj. Magnesii Sulfurici 20% (Polpharma S.A, Poland)		6	6	10	4
**Total volume**		mL	2500	2500	2500	2500
**Total energy**		kcal	1660	1910	1660	1660
